# Loneliness and smoking status: a pilot study of extended-stay hotel residents in Atlanta, Georgia

**DOI:** 10.3389/fpubh.2026.1770234

**Published:** 2026-05-08

**Authors:** Naomi Adjei, Terri Lewinson, Abhirupa Dasgupta, W. Moraa Onsando, Lauren A. Morgan

**Affiliations:** REACH Lab, The Dartmouth Institute for Health Policy and Clinical Practice, Dartmouth College, Geisel School of Medicine, Lebanon, NH, United States

**Keywords:** extended-stay hotel, gender, housing, loneliness, smoking status

## Abstract

**Introduction:**

As the housing industry struggles to meet growth demands in the United States, more people rely on extended-stay hotels (ESH) as their primary housing source. These settings often serve as a last resort for low-income families, many of whom face substandard housing quality and other conditions that can intensify environmental health risks. One complex environmental issue that disproportionately impacts ESH residents is tobacco smoke exposure. Although ESH residents understand the negative impacts of smoking on themselves and bystanders, previous qualitative research has found that ESH residents may view smoking as a shared activity that facilitates social engagement. However, other quantitative studies have demonstrated that smoking results in increased social isolation and loneliness. In this pilot study, we used multiple linear regression analyses to examine survey data from ESH residents, investigating the relationships between smoking status and loneliness.

**Methods:**

Data were obtained from a pilot study of ESH residents in Metropolitan Atlanta, Georgia. Participants completed a survey that included a demographic questionnaire, the De Jong Gierveld loneliness scale, and a smoking status questionnaire. A multiple regression analysis was conducted to investigate the degree to which smoking status is associated with loneliness.

**Results:**

The analysis based on a sample of 77 adults (aged 23–72) indicated that female smokers reported feeling lonely less often than male smokers in this sample (B = 1.10, *p* = 0.037). Additionally, men reported experiencing more social loneliness than women in this sample.

**Discussion:**

These results suggest that smoking status alone does not significantly predict loneliness in ESH residents. However, when smoking status is considered alongside gender, it substantially influences the loneliness experienced by ESH residents. Larger longitudinal samples are necessary to explore causal relationships between loneliness and smoking status in ESH residents across the adult lifespan.

## Introduction

Rising housing costs and a persistent shortage of affordable rental options have pushed many low-income and minoritized renters to rely on hotels as their primary residence, a trend increasingly common in metropolitan areas such as Atlanta (1–5). According to the National Low Income Housing Coalition, the U.S. is facing a shortage of 7.1 million affordable rental homes for extremely low-income renters, with only 35 homes available per 100 households nationwide (6). Furthermore, during the 2018–2019 school year, approximately 98,000 school children lived in hotels with their families, with estimates of 1 in 6 US children experiencing unstable housing in 2022 (7, 8). Extended-stay hotels (ESH) are increasingly functioning as long-term housing solutions rather than short-term accommodations, a trend that surged during the COVID-19 pandemic (9, 10). Given these statistics, research within this population is crucial to better understand their unique challenges and inform policies that support their psychological well-being.

Over the past two decades, researchers have extensively studied this population to better understand their unique living conditions as it relates to their physical and psychological well-being. Prior research indicates that individuals facing housing instability, such as ESH residents, have higher mortality rates, driven by substance dependence, mental and neurological illnesses, and infectious diseases, with drug dependence directly causing one-third of deaths (11). Furthermore, this population also experiences heightened vulnerability to other health risks, including those linked to tobacco use and exposure (12–15). Housing instability in childhood is linked to behavioral and mental health problems, and, combined with low preventive care and high absenteeism, may have lasting effects (16). In addition, ESH residents face heightened stressors related to housing instability and the threat of eviction, which contribute to an increased risk of mental health problems (17–22). To understand coping strategies related to housing instability, research concluded that residents primarily coped with their environment by changing their lifestyle to fit smaller spaces and by changing their perspective (23). Together, these findings and others emphasize the urgent need for interventions and policies that address both the structural conditions of housing instability and the health vulnerabilities faced by ESH residents.

Building on this need, a 2015 qualitative study examined perceptions of smoke exposure among 37 residents of extended-stay hotels in the Atlanta area, including 14 men and 23 women aged 19 to 64 years, of whom 65% identified as African American (15). Participants had lived in these hotels for varying lengths of time, ranging from two weeks to four years. The study found that both smokers and non-smokers viewed smoking as a social activity that facilitated connection and interaction with others, possibly due to a fundamental need to belong. Notably, these perceptions contrast with findings from research studies conducted in other populations, and they emphasize the bidirectional relationship between smoking and loneliness: loneliness can increase the likelihood of smoking as a coping mechanism, while smoking may, in turn, exacerbate feelings of loneliness by reinforcing social isolation or contributing to poor mental health (24–31).

Extending this line of research, data from the English Longitudinal Study of Ageing (ELSA) were used to examine the relationship between smoking, social isolation, and loneliness in adults aged 50 and older (*N* = 8,780) (27). In this study, participants provided self-reported data on smoking status and loneliness at baseline, with follow-up assessments conducted up to 12 years. The researchers hypothesized and concluded that, overall, smoking contributes to increased social isolation and heightened feelings of loneliness. Conversely, loneliness was associated with a greater risk of smoking, as well as other health-risk behaviors, in a study using the same ELSA database (*N* = 5,000) (31). To statistically examine the bidirectional relationship between loneliness and smoking, the first Mendelian randomization study employed inverse-variance weighted (IVW) regression and provided tentative evidence of a causal, bidirectional effect, particularly suggesting that smoking initiation may increase feelings of loneliness (28). These findings collectively highlight the complex, reciprocal relationship between loneliness and smoking, highlighting the need for interventions that address both social and behavioral determinants of health. This evidence is particularly relevant for populations such as ESH residents, whose experiences of housing instability, loneliness, and constrained living environments may amplify both loneliness and smoking-related health risks, emphasizing the importance of targeted strategies to support their well-being.

In this context, loneliness is defined as the subjective perception of a gap between the desired and actual number of social connections (social loneliness) or between the desired and actual level of intimate closeness (emotional loneliness) (32). Research asserts that individuals tend to experience greater emotional loneliness than social loneliness due to the greater distress caused by emotional loneliness (33–35). Overall, loneliness has been linked to various health issues such as increased anxiety and depression (36), higher risk factors for cardiovascular diseases (37), elevated stress (38), and a greater risk of dementia (39). Moreover, feelings of loneliness seem to be a more significant risk factor for drug and alcohol use and abuse (40–42). Additionally, smoking has been associated with deteriorating mental health conditions, as smokers face a higher risk for depression and anxiety (43, 44), creating a vicious cycle where both loneliness and smoking contribute to declining mental well-being. These interrelated effects of loneliness and other health risks emphasize the importance of investigating their relationship further, particularly in ESH residents, to inform interventions.

In this pilot study, we sought to explore the relationship between loneliness (dependent variables) and smoking behavior (independent variable) among ESH residents, a population in which smoking is prevalent. In addition, we sought to explore how gender modified the relationship based on previous studies suggesting that women tend to report greater loneliness than men (45–47). Consequently, due to the complexity of emotional loneliness in comparison to social loneliness (33–35, 48), we also sought to explore their relationship with smoking status. It is vital to understand how loneliness and smoking intersect in this specific group, as it could inform targeted interventions and health policies in ESH that promote safe smoking behaviors, reduce smoke exposure, and help smokers who may wish to begin their cessation journeys.

The purpose of this study was therefore to explore the associations between smoking status and loneliness among ESH residents, with gender tested as a moderator and age controlled for in the analyses. Specifically, the study aims to determine whether smoking status is significantly related to overall loneliness, as well as its emotional and social dimensions. It is hypothesized that smoking status will be positively associated with loneliness and that this relationship will be moderated by gender, such that the effect of smoking on loneliness will differ between men and women. Additionally, it is expected that among the two domains of loneliness, emotional loneliness will show a stronger positive association with smoking status than social loneliness, with gender as a moderating effect.

## Materials and methods

### Design

We employed a cross-sectional study design to explore the associations between psychological distress, as measured by loneliness, mental health, and depression, and smoking status. This study received ethical approval from the Dartmouth College Institutional Review Board, project #00032432. Participants completed all aspects of the study via Qualtrics. Before beginning the survey, participants were presented with an information sheet outlining the study’s purpose, procedures, and participation requirements.

### Survey questions

#### Basic characteristics of the population

Basic demographic characteristics were assessed using a Qualtrics questionnaire. Participants reported information including age, gender, race, ethnicity, income, length of stay in the hotel, and the number of adults and children residing in each room.

#### DeJong Gierveld Loneliness Scale

The DeJong Gierveld Loneliness Scale is a reliable six-item measure of overall, emotional, and social loneliness (32). It includes three statements on emotional loneliness and three on social loneliness, such as “There are enough people I feel close to.” Originally using a three-point scale, it was expanded to a five-point Likert scale. The scale differentiates social loneliness, characterized by fewer social relationships than desired, from emotional loneliness, which reflects unmet needs for intimacy in close relationships. Scoring assigns 1 point to positive/neutral responses for questions 1–3 and negative/neutral responses for questions 4–6, while negative and positive responses, respectively, receive 0 points. The total score ranges from 0 (least lonely) to 6 (most lonely). The Cronbach’s alpha for this scale typically ranges between 0.70 and 0.76; in this study, it was 0.73, indicating an acceptable consistency among the items.

#### Smoking status

Smoking status was assessed through self-reported responses based on questions derived from a validated survey, the Behavioral Risk Factor Surveillance System (BRFSS) (49). The survey posed the question, “What is your current smoking status?” Response options included “never smoked,” “quit all smoking three months ago,” “quit tobacco but currently using smokeless tobacco or E-cigarettes,” and “current smoker.” Participants who answered “never smoked” or “quit all smoking three months ago” were assigned a score of 0 (non-smoker). Those who responded with “quit tobacco but currently using smokeless tobacco or E-cigarettes” or “current smoker” were assigned a score of 1 (smoker).

### Procedure

The measures included in this paper are part of a larger pilot study on tobacco smoking in ESH (12). The sample included 80 participants recruited from ESHs in Metropolitan Atlanta, Georgia, through a respondent-driven sample (RDS) method (50). Recruitment efforts included the distribution of flyers containing information about the study, a research team contact email for questions, and a QR code linking directly to the survey. Participants were also recruited through paid referrals, allowing individuals to refer others to the study in exchange for compensation. Eligibility criteria included age 18 or older, English proficiency, being able to consent, and identifying as long-term renters in Atlanta ESHs (defined as renting consecutively for at least 3 weeks). Per Title 45 of the Code of Federal Regulations, subsection entitled General requirements for informed consent (45 CFR 46.116(f)), signed consent was not necessary for the completion of this web-based, anonymous survey (51).

Participants were informed that continuing with the survey would constitute their consent to participate in the study, provided they had been informed of its purpose, scope, and risks. They were also advised to close their browser window if they did not wish to participate. Eligible participants then completed questionnaires assessing demographic information, the DeJong Gierveld Loneliness Scale, and self-reported measures of smoking status. Surveys were self-administered and comprised of self-reported measures distributed via Qualtrics.

### Statistical analysis

All analyses were conducted using Stata version 17.0 (StataCorp LLC, College Station, TX, United States). Descriptive statistics, including means, standard deviations, and ranges, were calculated for all variables, such as loneliness scores, smoking status, mental health indicators, and demographic characteristics, including age, gender, race, and ethnicity. Gender was coded as female = 1 and male = 0. Chronological age was measured in years, and household size was calculated by summing the number of adults and children residing in each hotel room.

Multiple linear regression analyses were performed to examine the association between smoking status and overall loneliness. In Model 1, the association between smoking status and loneliness was examined without covariates. Model 2 adjusted for age in this association. Model 3 adjusted for both age and gender. Model 4 included an interaction term between smoking status and gender to assess whether gender moderated the association.

Additional multiple linear regression analyses were used to examine the relationship between smoking status and the specific domains of loneliness, emotional loneliness, and social loneliness. Two separate datasets were created for these analyses. In the emotional loneliness dataset, Model 1 examined the unadjusted association between emotional loneliness and smoking status. Model 2 adjusted the association for age, while Model 3 further adjusted for both age and gender. Model 4 included an interaction term between smoking status and gender. The same sequence of models was applied to the social loneliness dataset, with each model testing the association between smoking status and social loneliness under increasing levels of adjustment and finally assessing gender moderation in Model 4.

This structured modeling approach allowed for a comprehensive examination of the relationships between smoking status and loneliness across multiple dimensions, while also assessing whether gender moderated these associations.

### Diagnostic tests and assumption checks

To ensure the validity of our multiple linear regression models, we conducted several diagnostic tests. Normality of residuals was assessed using the Shapiro–Wilk test and visual inspection of Q–Q and P–P plots, as well as histograms. Multicollinearity among predictors was evaluated using Variance Inflation Factors (VIF). These procedures were applied to all regression models to confirm that the assumptions of linearity, homoscedasticity, and independence were reasonably satisfied.

## Results

### Sample description

The analytical sample consisted of participants with valid responses for all study variables, totaling 77 out of 80 survey respondents who identified as long-term renters in Atlanta ESHs (defined as renting consecutively for at least three weeks). Participants ranged in age from 23 to 72 years (M = 42.31, SD = 11.11; see Table 1). The sample was predominantly composed of non-Hispanic Black/African American females (see Table 1). Multicollinearity diagnostics indicated no concerns among the independent variables. Variance inflation factors were very low (VIFs = 1.01–1.02; mean VIF = 1.01), suggesting that age, gender assigned at birth, and smoking status were not highly correlated.

**Table 1:** Descriptive statistics for study variables.

Variable	*N*	Mean	SD	Min	Max
Loneliness	77	3.51	1.28	1	6
Smoking status	77	0.55	0.5	0	1
Age	77	42.31	11.11	23	72
Gender – female	52	0.68	0.47	0	1
Race-Black	57	0.69	0.47	0	1
Ethnicity-Non-Hispanic/Latino/Cuban	58	0.08	0.27	0	1

### The relationship between smoking status and loneliness, with gender as a moderator

**Figure 1:**
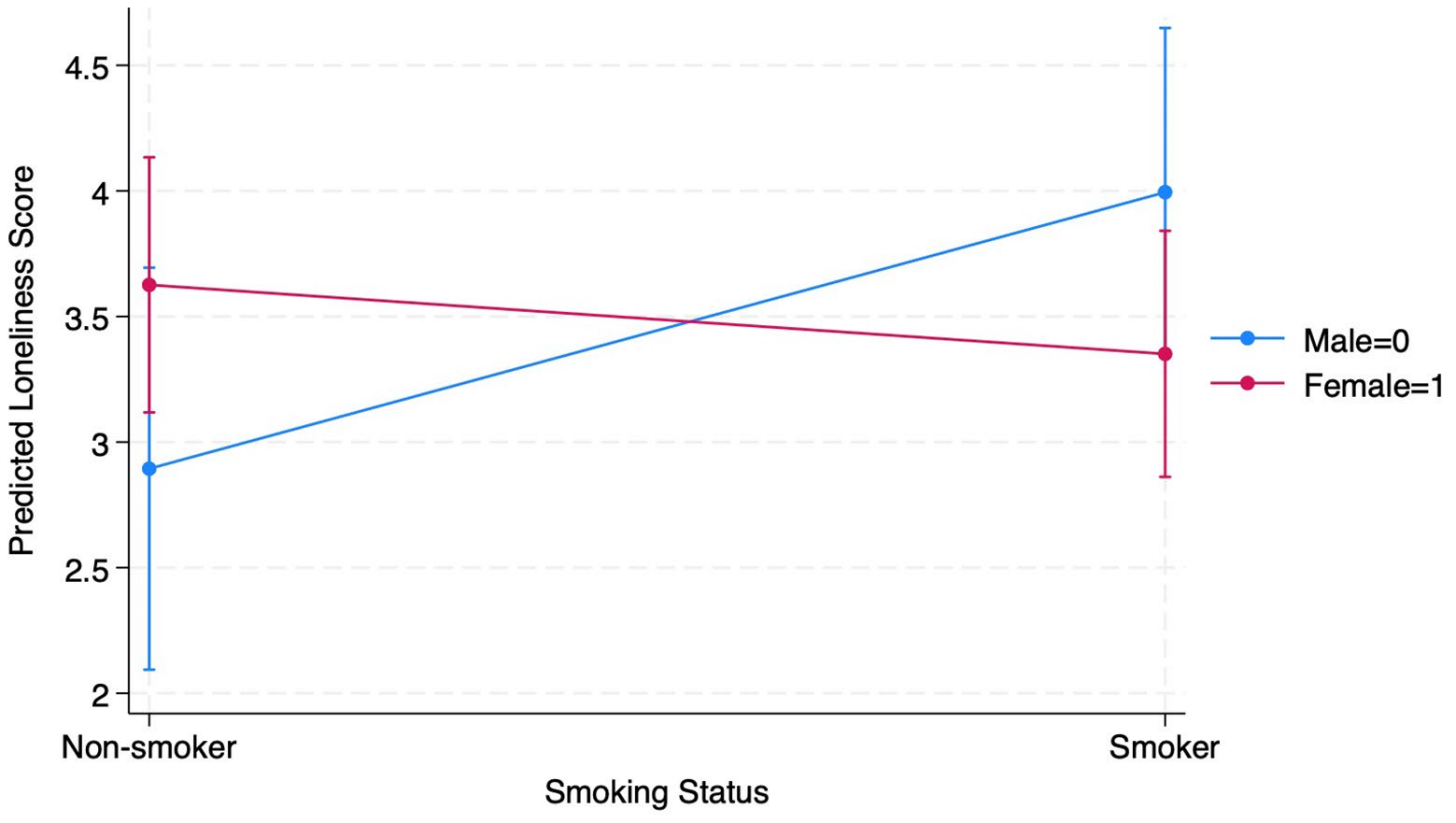
Gender as a moderator between loneliness and smoking.

Based on this multiple linear regression analysis, model 1 indicated that smoking status did not significantly explain variations in the total loneliness score (see Supplementary data for full analyses). Thus, smoking status appeared to have little to no effect on loneliness in this sample (see Table 2; *B* = 1.43, *p* = 0.639). The inclusion of covariates age and gender in models 2 and 3 suggested that neither smoking status, age, nor gender, individually or together, was a significant predictor of loneliness (see Table 2). Model 4 included a fully adjusted model that included an interaction term between smoking status and gender to examine the moderation effect of gender on loneliness and smoking status. In model 4, smoking status is the most significant predictor, with smokers reporting higher loneliness scores compared to non-smokers (B = 1.101, *p* = 0.037). However, age and gender did not have significant effects. The significant interaction between smoking status and gender suggested that the relationship between smoking and loneliness is moderated by gender (B = −1.38, *p* = 0.032). Specifically, smoking had a different impact on loneliness depending on whether an individual is male or female. For females who smoke, the loneliness score is 1.38, lower than expected compared to males, which suggests that the positive relationship between smoking and loneliness is reduced for females (see Figure 1).

**Table 2:** Multiple linear regression analysis: loneliness and smoking status.

Variable	Model 1 B/(SE)	Model 2 B/(SE)	Model 3 B/(SE)	Model 4 B(SE)
Smoking status	0.143/(0.295)	0.169/(0.297)	0.165/(0.300)	1.101*/(0.518)
Age		0.012/(0.013)	0.011/(0.014)	0.008/(0.013)
Gender			−0.056/(0.318)	0.732/(0.475)
Interaction model Smoking × Gender				−1.376*/(0.628)
Constant	3.428***/(0.218)	2.950***/(0.625)	2.995***/(0.679)	2.526***/(0.696)
Model *p*-value	0.630	0.639	0.821	0.299

DV: Loneliness. Bold values indicate statistically significant results (*p* < 0.05). Significance levels are denoted as *p < 0.001; ***p < 0.05. This table represents a multiple linear regression analysis performed to examine the extent to which smoking status is associated with loneliness, using overall loneliness score as the dependent variable and smoking status as the independent variable. Model 1 included an unadjusted association between loneliness and smoking status. Model 2 adjusted loneliness and smoking status for age. Model 3 adjusted loneliness and smoking status for both age and gender. Model 4 included an interaction term between smoking status and gender.

### The relationship between smoking status and emotional loneliness, with gender as a moderator

Based on this multiple regression analysis, model 1 indicated that smoking status did not significantly explain variations in the emotional loneliness score (see Supplementary data for full analyses). Thus, smoking status seemed to have a marginal effect on emotional loneliness in this sample (see Table 3; B = 0.126, *p* = 0.072). The inclusion of covariates age and gender in modules 2 and 3 suggested that neither smoking status, age, nor gender, individually or together, was a significant predictor of emotional loneliness (see Table 4 and Supplementary data for full analyses). Model 4 included a fully adjusted model that included an interaction term between smoking status and gender to examine the moderation effect of gender on emotional loneliness and smoking status. The overall model indicates significance at *p* = 0.007. Smoking status was the most significant predictor, with smokers reporting higher loneliness scores compared to non-smokers (B = 1.40, *p* < 0.001).

**Table 3:** Multiple regression analysis: emotional loneliness and smoking status.

Variable	Model 1 B/(SE)	Model 2 B/(SE)	Model 3 B/(SE)	Model 4 B(SE)
Smoking status	0.126/(0.021)	0.125/(0.021)	0.122/(0.021)	1.400***/(0.518)
Age		−0.004/(0.011)	−0.005/(0.011)	0.002/(0.011)
Gender			−0.043/(0.017)	1.158**/(0.017)
Interaction model Smoking × Gender				−1.398**/(0.485)
Constant	1.229***/(0.168)	1.085***/(0.483)	0.798***/(0.679)	2.526***/(0.696)
Model *p*-value	0.072	0.191	0.141	0.007**

DV: Emotional Loneliness. Bold values indicate statistically significant results (*p* < 0.05). Significance levels are denoted as **p < 0.01; ***p < 0.05. This table represents a multiple linear regression analysis to examine the extent to which smoking status is associated with emotional loneliness, using emotional loneliness score as the dependent variable and smoking status as the independent variable. Model 1 included an unadjusted association between emotional loneliness and smoking status. Model 2 adjusted for emotional loneliness and smoking status for age. Model 3 adjusted for emotional loneliness and smoking status for both age and gender. Model 4 included an interaction term between smoking status and gender.

**Table 4:** Multiple regression analysis: social loneliness and smoking status.

Variable	Model 1 B/(SE)	Model 2 B/(SE)	Model 3 B/(SE)	Model 4 B(SE)
Smoking status	−0.271/(0.194)	−0.252/(0.196)	−0.284/(0.193)	−0.299/(0.344)
Age		0.007/(0.0009)	−0.005/(0.009)	−0.007/(0.009)
Gender			−0.414/(0.017)*	−0.426/(0.315)
Interaction model Smoking × Gender				0.007/(0.039)
Constant	2.200***/(0.144)	1.866***/(0.413)	2.263***/(0.184)	2.158***/(0.696)
Model *p*-value	0.168	0.268	0.085	0.079

DV: Social Loneliness. Bold values indicate statistically significant results (*p* < 0.05). Significance levels are denoted as *p < 0.001; ***p < 0.05. This table represents a multiple linear regression analysis to examine the extent to which smoking status is associated with social loneliness, using social loneliness score as the dependent variable and smoking status as the independent variable. Model 1 included an unadjusted association between social loneliness and smoking status. Model 2 adjusted for social loneliness and smoking status by age. Model 3 adjusted social loneliness and smoking status for both age and gender. Model 4 included an interaction term between smoking status and gender.

**Figure 2:**
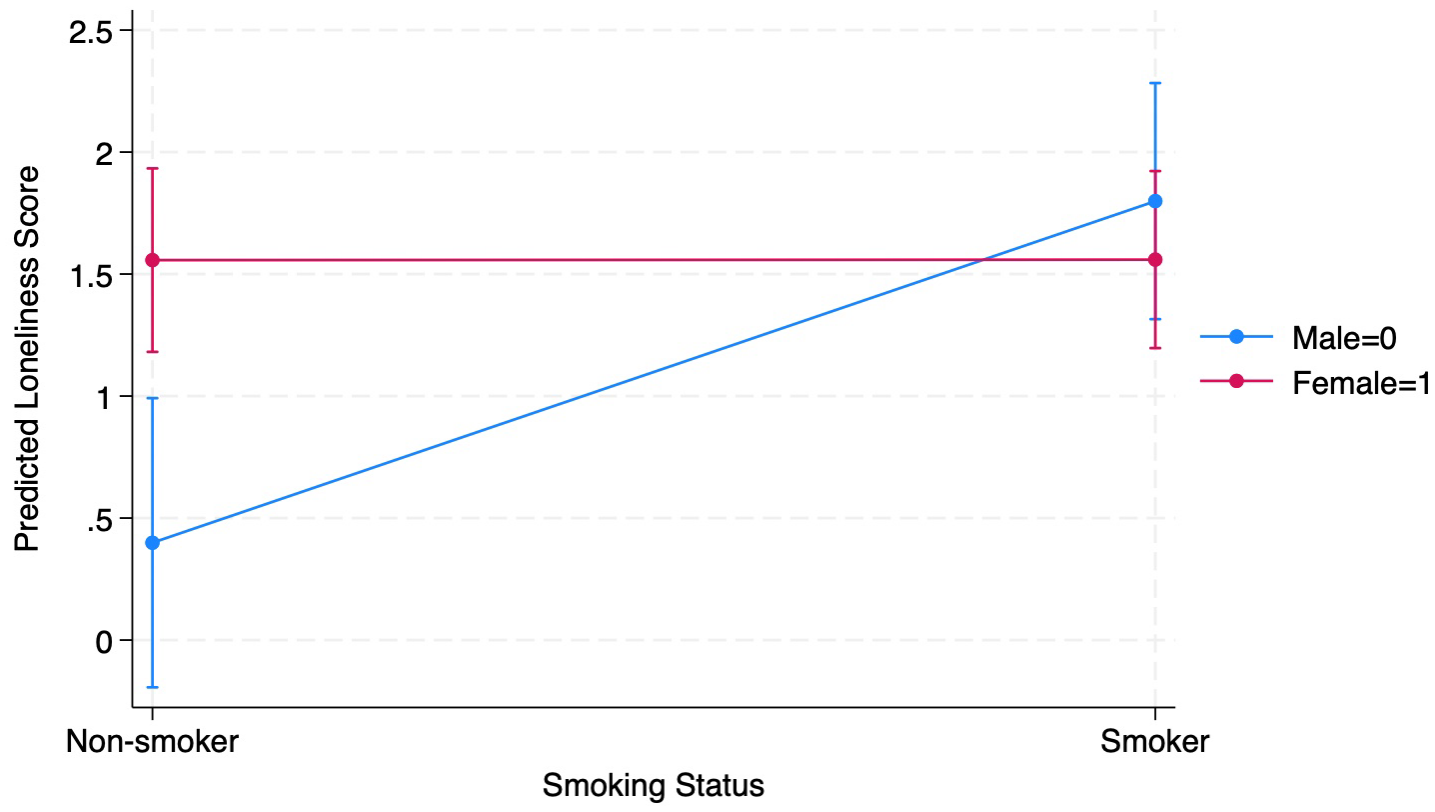
Gender as a moderator between emotional loneliness and smoking.

Furthermore, gender also showed a significant positive relationship with emotional loneliness in this model (B = 1.40, *p* = 0.002). This indicates that females are predicted to report greater emotional loneliness compared to males in this sample. However, age did not indicate a significant effect. The significant interaction between smoking status and gender suggests that the relationship between smoking and loneliness is moderated by gender (B = −1.39, *p* = 0.004). Specifically, the positive impact of smoking on emotional loneliness is reduced for females, such that female smokers are less likely to report emotional loneliness than male smokers (see Figure 2).

### The relationship between smoking status and social loneliness, with gender as a moderator

This multiple regression analysis consistently indicated across all models that smoking status and age did not provide strong evidence of being significant predictors of loneliness (see Supplementary data for full analyses). Model 3 revealed that gender, on its own, significantly impacted social loneliness in the analysis (see Table 4; B = 2.30, *p* = 0.046). Gender had a negative relationship with social loneliness (B = −0.4137), suggesting that females were more likely to report lower levels of social loneliness compared to males. This relationship is statistically significant (*p* = 0.046), indicating that gender indeed had a significant effect on social loneliness in this model. However, gender lost its significance in the interaction model (smoking status*gender).

Diagnostic checks indicated that residuals were approximately normally distributed, with a Shapiro–Wilk W = 0.963 (*p* = 0.026). Visual inspection of Q–Q and P–P plots, as well as a histogram of residuals, suggested only minor deviations from normality at the tails. Variance Inflation Factors for all predictors were near 1 (mean VIF = 1.01), indicating minimal multicollinearity.

## Discussion

There is a growing body of literature exploring the physical and psychological well-being of ESH residents to better understand their unique living conditions, with particular attention to factors that drive smoking behavior among this population (9–14). In this study of ESH residents in Metropolitan Atlanta, we set out to explore the relationship between smoking status and loneliness, including emotional and social domains, with gender as a potential moderator. Loneliness was assessed using the DeJong Gierveld Loneliness Scale, while smoking status was assessed through self-reported responses based on questions derived from BRFSS. The sample was non-random and recruited from a limited number of ESH sites in Atlanta, Georgia, which may limit generalizability. The demographic composition of the sample was compared with available national data on hotel-based housing instability, and key characteristics (e.g., income range, racial/ethnic composition) were broadly comparable, though not identical (52, 53). Overall, the results suggest that the relationship between smoking and loneliness is nuanced and varies depending on the type of loneliness examined. Diagnostic checks supported the adequacy of the regression model and suggest that the reported associations are unlikely to be biased by violations of key assumptions.

For overall loneliness, smoking status alone did not significantly predict loneliness scores. This suggests that smoking had little to no effect on loneliness in this sample, regardless of age and gender. Although these results differed from our hypothesis and prior quantitative findings that suggested a positive relationship (27), they align with previous qualitative research indicating that smoking may serve as a form of social connection within this population (15). Differences in findings may be related to variations in sample size or to the unique living conditions of ESH residents, which may foster adaptive behaviors for survival, such as social belonging. However, the inclusion of gender as a moderator revealed that smoking was associated with higher loneliness, but this effect was decreased for females. Specifically, female smokers reported lower loneliness scores than expected relative to male smokers, indicating that the impact of smoking on loneliness differs by gender. Specifically, female smokers reported lower loneliness scores than male smokers, suggesting that male smokers in this sample experienced greater loneliness than their female counterparts. These results were counterintuitive to previous research, which asserted that women tend to report greater overall loneliness than men (45–47). One possible explanation for this unexpected finding is that the relationship between smoking and loneliness may vary by gender within this population. For example, men who smoke may encounter different social or psychological factors contributing to loneliness than women, such as social stigma or distinct coping strategies. Additionally, the way loneliness is experienced or reported by men in this study may differ from patterns documented in previous research, highlighting the complexity of how smoking and gender intersect to shape loneliness. Although some studies have begun to examine gendered differences in loneliness, our results emphasize the importance of developing interventions for ESH residents that address smoking and loneliness in ways that are responsive to gender-specific needs. This is particularly relevant given the extant evidence of disparities in secondhand smoke exposure in home and vehicle environments, including among marginalized gender groups in multi-unit or unstable housing settings, which mirrors the conditions faced by many ESH residents (54–56).

When examining emotional loneliness, a similar but more pronounced pattern emerged. Smoking status significantly predicted higher emotional loneliness, and females overall reported higher emotional loneliness than males. Importantly, the interaction between smoking and gender indicated that female smokers were less likely to experience emotional loneliness compared to male smokers. This suggests that the emotional consequences of smoking may be more detrimental for males in this population, while females may benefit from protective factors, such as stronger social support networks or adaptive coping strategies, that mitigate the impact of smoking. These findings highlight the differences in emotional and social loneliness related to smoking status among ESH residents and align with previous research showing that, due to its complexity, emotional loneliness is typically more pronounced than social loneliness (33–35).

In contrast, for social loneliness, smoking status did not emerge as a significant predictor, and age also had no notable effect. Gender, however, independently predicted social loneliness, with females reporting lower levels than males. Which is consistent with other observations in diverse contexts (57–60). This suggests that female residents in ESHs may have stronger social networks or more frequent social interactions, contributing to lower social loneliness. Notably, when the interaction term between smoking and gender was included, the moderating effect was no longer significant, indicating that smoking does not differentially affect social loneliness by gender. This trend in gender differences in social loneliness reflects a broader trend rather than being unique to extended-stay hotel (ESH) residents. Research by the American Survey Center concluded that men’s social circles are shrinking, with 55% of men reporting having six or more close friends in 1999, compared to only 27% today (61). Thus, interventions targeting social loneliness could focus on strategies to enhance men’s psychological well-being. Additionally, male smokers appear to experience greater emotional loneliness, highlighting the complex psychological effects of smoking (25, 62).

### Public health implications

While these findings offer preliminary insight, their implications for policy and intervention should be interpreted cautiously until they are confirmed in larger and more representative samples. Nevertheless, addressing smoking behaviors among ESH residents may benefit from a gender-sensitive approach. Public health interventions could incorporate gender-responsive strategies, such as support groups and counseling services tailored to the unique experiences, stressors, and triggers faced by male and female residents. Policies and implementation strategies within ESH residents should explicitly consider gender dynamics, ensuring that smoking cessation resources are accessible, culturally appropriate, and effective for all residents. By integrating gender-sensitive approaches, interventions can more effectively reduce smoking-related health risks and mitigate associated loneliness among residents.

### Strengths and limitations

This exploratory pilot study contributes to the existing literature on smoking and loneliness by offering a novel perspective on ESH residents. To the best of our knowledge, this is the first research study to explore loneliness, mental health, and smoking behavior in ESH residents. The insights from this study are valuable because they identify interesting relationships between smoking status, loneliness, mental health, and gender that could inform targeted interventions and support programs tailored to the needs of ESH residents.

The findings of this study should be considered in light of its limitations, particularly the small sample size of 77 participants. Additionally, Black individuals and women made up most of the sample, with less representation of men, Hispanic individuals, and individuals of other races. While respondent-driven sampling was employed in later recruitment waves, much of our sample was recruited via convenience sampling and thus, likely included volunteer bias. Finally, our geographical scope was limited to the metropolitan Atlanta area. These limitations resulted in insufficient statistical power to detect significant relationships between smoking and loneliness that can be generalized to individuals who use extended-stay hotels as their primary residence. However, future projects should aim to use larger, longitudinal samples to improve power and investigate these trends in the population of ESH residents at large.

An additional limitation is that the present pilot survey did not directly measure specific housing environmental characteristics, such as room size, ventilation conditions, or overall housing quality within extended-stay hotels. These factors may influence environmental health risks and resident satisfaction, particularly in shared living spaces where tobacco smoke exposure may occur. Future research should incorporate direct measures of housing conditions, including ventilation, spatial density, and environmental quality, to better understand how these contextual factors interact with smoking behaviors and psychosocial outcomes among ESH residents.

## Conclusion

ESH residents may experience complex psychological challenges, including loneliness, due to the transient and often unpredictable nature of their living environments. This study examined the relationships between smoking status and loneliness among ESH residents and found patterns that align with prior qualitative research suggesting that smoking may function as a form of social connection rooted in a fundamental need to belong.

Overall, findings indicate that smoking status alone does not significantly predict loneliness; however, when gender is considered, important differences emerge. Female smokers reported lower levels of loneliness than male smokers, suggesting that gender meaningfully shapes the smoking-loneliness relationship. Additionally, men in this sample reported higher levels of social loneliness than women, highlighting potential gender disparities in social support or connectedness within ESH settings.

Although the association between smoking and emotional loneliness was only marginally significant across models, smoking consistently emerged as the strongest predictor of loneliness, while demographic factors such as age and gender played relatively minimal direct roles. These findings emphasize the importance of considering both gender and the type of loneliness being experienced when developing interventions to support the well-being of ESH residents.

Future research should replicate these findings in larger, more diverse samples and examine potential mechanisms, such as social support, coping strategies, or stress levels, that might explain why gender moderates the impact of smoking on loneliness. Additionally, longitudinal studies could clarify causal relationships between smoking behavior and loneliness over time.

## Data Availability

The original contributions presented in the study are included in the article/Supplementary material, further inquiries can be directed to the corresponding author.
