# Supplementary material for: Loneliness and smoking status: a pilot study of extended-stay hotel residents in Atlanta, Georgia
Source: Front Public Health. 2026 May 8;14:1770234. doi: 10.3389/fpubh.2026.1770234 (PMC13194354; doi:10.3389/fpubh.2026.1770234)
Supplement: Supplementary file 1 [file Table_1.docx]

**Supplemental Tables:**

*Results of Two-Sample t-Test for DeJong Loneliness Total Score by Smoking Status*

| **Group** | **N** | **Mean** | **Std. Error** | **Std. Deviation** | **95% Confidence Interval** |
| --- | --- | --- | --- | --- | --- |
| Non-smokers (0) | 35 | 3.43 | 0.21 | 1.22 | [3.01, 3.85] |
| Smokers (1) | 42 | 3.57 | 0.21 | 1.35 | [3.15, 3.99] |
| Combined | 77 | 3.51 | 0.15 | 1.28 | [3.22, 3.80] |

**Note.**
*Diff* = Mean(0) − Mean(1) = -0.14, Standard Error = 0.30, 95% CI = [-0.73, 0.45].
t(75) = -0.48, p = 0.315 (two-tailed).
The null hypothesis (H₀: diff = 0) was tested. Pr(T < t) = 0.315, Pr(T > t) = 0.685.

*Linear Regression Model of Loneliness and Smoking Status*

| **Variable** | **B** | **SE** | ***t*** | **p** | **[95% Confidence Interval]** |
| --- | --- | --- | --- | --- | --- |
| Smoking Status | 0.143 | 0.295 | 0.48 | 0.630 | [-0.445, 0.731] |
| Constant | 3.428 | 0.218 | 15.72 | 0.000 | [2.994, 3.863] |
| F(1, 75) = 0.23, R² =0.003,  Adj. R² = -0.01, p=0.639 |  |  |  |  |  |

**DV: Loneliness**

*p < .05; **p < .01; ***p < .001

*Linear Regression Model of Loneliness and Smoking Status, Age as Covariates*

| **Variable** | **B** | **SE** | ***t*** | **p** | **95% Confidence Interval** |
| --- | --- | --- | --- | --- | --- |
| Smoking Status | 0.169 | 0.297 | 0.55 | 0.58 | [-0.43, 0.76] |
| Age | 0.012 | 0.013 | 0.82 | 0.42 | [-0.02, 0.04] |
| Constant | 2.950 | 0.625 | 4.72 | 0.00 | [1.70, 4.20] |
| F(2, 74) = 0.45, = R² = 0.012,  Adj. R² = -0.015, p=0.639 |  |  |  |  |  |

**DV: Loneliness**

*p < .05; **p < .01; ***p < .001

*Linear Regression Model of Loneliness and Smoking Status, Age and Gender as Covariates*

| **Variable** | **B** | **SE** | ***t*** | ***p*** | **95% Confidence Interval** |
| --- | --- | --- | --- | --- | --- |
| Smoking Status | 0.165 | 0.300 | 0.55 | 0.584 | [-0.434, 0.765] |
| Age | 0.011 | 0.014 | 0.80 | 0.426 | [-0.016, 0.037] |
| Gender | -0.056 | 0.318 | -0.18 | 0.861 | [-0.690, 0.577] |
| Constant | 2.995 | 0.679 | 4.41 | 0.000 | [1.641, 4.349] |
| F(1, 75) = 0.31, R² =0.003,  Adj. R² = -0.028, p=0.821 |  |  |  |  |  |

**DV: Loneliness**

*p < .05; **p < .01; ***p < .001

*Linear Regression Analysis of Loneliness and Smoking Status, with the*

*Interaction Term (Smoking Status and Gender)*

| **Variable** | **B** | **SE** | ***t*** | ***p*** | **95% Confidence Interval** |
| --- | --- | --- | --- | --- | --- |
| Smoking Status | 1.101 | 0.518 | 2.13 | 0.037 | [0.068, 2.133] |
| Age | 0.008 | 0.013 | 0.66 | 0.513 | [-0.177, 0.035] |
| Gender | 0.732 | 0.475 | 1.54 | 0.128 | [-0.215, 1.678] |
| Smoking Status × Gender | -1.376 | 0.628 | -2.19 | 0.032 | [-2.628, -0.124] |
| Constant | 2.526 | 0.696 | 3.63 | 0.001 | [1.138, 3.914] |
| F(2, 72) = 1.44, R² =0.074,  Adj. R² = 0.023, p=0.229 |  |  |  |  |  |

**DV: Loneliness**

*p < .05; **p < .01; ***p < .001

*Linear Regression Model of Emotional Loneliness and Smoking Status*

| **Variable** | **B** | **SE** | ***t*** | ***p*** | **95% Confidence Interval** |
| --- | --- | --- | --- | --- | --- |
| Smoking Status | 0.414 | 0.227 | 1.82 | 0.630 | [-0.038, 0.73] |
| Constant | 1.228 | 0.167 | 7.33 | 0.000 | [2.99, 3.86] |
| F(1, 75) = 3.33, R² =0.072,  Adj. R² = 0.029, p=0.072 |  |  |  |  |  |

**DV: Emotional Loneliness**

*p < .05; **p < .01; ***p < .001

*Linear Regression Model of Emotional Loneliness and Smoking Status, Age as Covariates*

| **Variable** | **B** | **SE** | ***t*** | ***p*** | **95% Confidence Interval** |
| --- | --- | --- | --- | --- | --- |
| Smoking Status | 0.422 | 0.229 | 1.84 | 0.070 | [-0.356, 0.880] |
| Age | 0.003 | 0.010 | 0.32 | 0.751 | [-0.017, 0.024] |
| Constant | 1.084 | 0.483 | 2.25 | 0.028 | [1.22, 2.047] |
| F(2, 74) = 1.70, R² =0.044,  Adj. R² = 0.012, p=0.190 |  |  |  |  |  |

**DV: Emotional Loneliness**

*p < .05; **p < .01; ***p < .001

*Linear Regression Model of Emotional Loneliness and Smoking Status, Age and Gender as Covariates*

| **Variable** | **B** | **SE** | ***t*** | ***p*** | **95% Confidence Interval** |
| --- | --- | --- | --- | --- | --- |
| Smoking Status | 0.449 | 0.228 | 1.97 | 0.053 | [-0.006, 0.905] |
| Age | 0.004 | 0.010 | 0.39 | 0.698 | [-0.016, 0.025] |
| Gender | 0.357 | 0.242 | 1.48 | 0.144 | [-0.125, 0.839] |
| Constant | 0.797 | 0.516 | 1.54 | 0.127 | [-0.232, 1.827] |
| F(3, 73) = 2.30, R² =0.0836,  Adj. R² = 0.049, p=0.085 |  |  |  |  |  |

**DV: Emotional Loneliness**

*p < .05; **p < .01; ***p < .001

*Linear Regression Model of Emotional Loneliness and Smoking Status, with the*

*Interaction Term (Smoking Status and Gender)*

| **Variable** | **B** | **SE** | ***t*** | ***p*** | **95% Confidence Interval** |
| --- | --- | --- | --- | --- | --- |
| Smoking Status | 1.400 | 0.383 | 3.65 | 0.000 | [0.635, 2.165] |
| Age | 0.002 | 0.009 | 0.19 | 0.852 | [-0.017, 0.021] |
| Gender | 1.158 | 0.352 | 3.29 | 0.002 | [0.457, 1.859] |
| Smoking Status × Gender | -1.397 | 0.465 | -3.00 | 0.004 | [-2.325, -0.471] |
| Constant | 0.32 | 0.52 | 0.62 | 0.535 | [-0.71, 1.35] |
| F(4, 72) = 3.82, R² =0.175,  Adj. R² = 0.129, p=0.0072 |  |  |  |  |  |

**DV: Emotional Loneliness**

*p < .05; **p < .01; ***p < .001

*Linear Regression Model of Social Loneliness and Smoking Status*

| **Variable** | **B** | **SE** | ***t*** | ***p*** | **[95% Confidence Interval]** |
| --- | --- | --- | --- | --- | --- |
| Smoking Status | -0.271 | 0.194 | -1.39 | 0.168 | [-0.659, 0.117] |
| Constant | 2.200 | 0.144 | 15.28 | 0.000 | [1.913, 2.486] |
| F(1, 75) = 1.94, R² =0.025,  Adj. R² = 0.012, p=0.168 |  |  |  |  |  |

**DV: Social Loneliness**

*p < .05; **p < .01; ***p < .001

*Linear Regression Model of Social Loneliness and Smoking Status, Age as Covariates*

| **Variable** | **B** | **SE** | ***t*** | ***p*** | **95% Confidence Interval** |
| --- | --- | --- | --- | --- | --- |
| Smoking Status | -0.253 | 0.196 | -1.29 | 0.203 | [-0.644, 0.139] |
| Age | 0.008 | 0.009 | 0.86 | 0.390 | [-0.009, 0.0253] |
| Constant | 1.865 | 0.413 | 4.52 | 0.000 | [1.044, 2.688] |
| F(2, 74) = 1.34, R² =0.035,  Adj. R² = 0.009, p=0.268 |  |  |  |  |  |

**DV: Social Loneliness**

*p < .05; **p < .01; ***p < .001

*Linear Regression Model of Social Loneliness and Smoking Status, Age and Gender as Covariates*

| **Variable** | **B** | **SE** | ***t*** | ***p*** | **95% Confidence Interval** |
| --- | --- | --- | --- | --- | --- |
| Smoking Status | -0.284 | 0.193 | -1.47 | 0.145 | [-0.669, 0.101] |
| Age | 0.007 | 0.009 | 0.79 | 0.435 | [-0.010, 0.024] |
| Gender | -0.413 | 0.204 | -2.03 | 0.046 | [-0.821, 0.007] |
| Constant | 2.198 | 0.436 | 5.04 | 0.000 | [1.328, 3.07] |
| F(3, 73) = 2.30, R² =0.086,  Adj. R² = 0.049, p=0.085 |  |  |  |  |  |

**DV: Social Loneliness**

*p < .05; **p < .01; ***p < .001

*Linear Regression Model of Social Loneliness and Smoking Status, with the*

*Interaction Term (Smoking Status and Gender)*

| **Variable** | **B** | **SE** | ***t*** | | ***p*** | | **95% Confidence Interval** |
| --- | --- | --- | --- | --- | --- | --- | --- |
| Smoking Status | -0.299 | 0.344 | -0.87 | | 0.387 | | [-0.983, 0.385] |
| Age | 0.007 | 0.008 | 0.78 | | 0.437 | | [-0.010, 0.024] |
| Gender | -0.426 | 0.315 | -1.35 | | 0.180 | | [-1.054, 0.201] |
| Smoking Status × Gender | 0.022 | 0.417 | 0.05 | | 0.958 | | [-0.808, 0.852] |
| (Constant) | 2.205 | 0.462 | 4.78 | | 0.000 | | [1.285, 3.125] |
| F(3, 73) = 2.30, R² =0.086,  Adj. R² = 0.049, p=0.085 |  |  |  |  | |  | |

**DV: Social Loneliness**

*p < .05; **p < .01; ***p < .001

**DeJong Gierveld Loneliness Scale**

In this 6-item scale, three statements are made about ‘emotional loneliness’ and three about ‘social loneliness’. Social loneliness (SL) occurs when someone is missing a wider social network and emotional loneliness (EL) is caused when you miss an “intimate relationship”.

1. I experience a general sense of emptiness [EL]


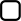
 Yes


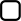
 More or less


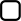
 No 2. I miss having people around me [EL]


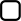
 Yes


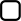
 More or less


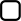
 No

1. I often feel rejected [EL]


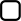
 Yes


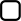
 More or less


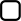
 No

1. There are plenty of people I can rely on when I have problems [SL]


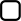
 Yes


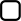
 More or less


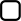
 No

1. There are many people I can trust completely [SL]


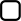
 Yes


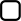
 More or less


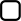
 No

1. There are enough people I feel close to [SL]


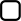
 Yes


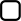
 More or less
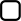
 No
