# Supplementary material for: Loneliness and smoking status: a pilot study of extended-stay hotel residents in Atlanta, Georgia
Source: Front Public Health. 2026 May 8;14:1770234. doi: 10.3389/fpubh.2026.1770234 (PMC13194354; doi:10.3389/fpubh.2026.1770234)
Supplement: Supplementary file 2 [file Supplementary_file_1.docx]

FIG 1: Normal q-q Plot of Age

Fig 2: Histogram of Residuals with Normal Curve

Fig 3: Normal Q–Q Plot of Residuals

Fig 4: Normal P–P Plot of Residuals

Table 1: Variance Inflation Factor (VIF) for Predictor Variables

| **Variable** | **VIF** | **Interpretation** |
| --- | --- | --- |
| Smoking Status | 1.02 | No multicollinearity |
| Age | 1.01 | No multicollinearity |
| Gender | 1.01 | No multicollinearity |
| **Mean VIF** | **1.01** | Excellent |

*Note: Multicollinearity diagnostics indicated no concerns among the independent variables. Variance inflation factors were very low (VIFs = 1.01–1.02; mean VIF = 1.01), suggesting that age, gender assigned at birth, and smoking status were not highly correlated.*

Table 2: Shapiro–Wilk Test for Normality of Regression Residuals

| **Variable** | **Obs** | **W** | **V** | **z** | **Prob > z** |
| --- | --- | --- | --- | --- | --- |
| Residual | 77 | 0.96344 | 2.432 | 1.943 | 0.02601 |

*Note: The Shapiro–Wilk test assesses whether residuals are normally distributed. A p-value < .05 indicates a statistically significant deviation from normality.*
